# Thermally Activated on‐Surface Self‐Metalation of Pd‐Phthalocyanines

**DOI:** 10.1002/chem.202500944

**Published:** 2025-04-18

**Authors:** Mattia Bassotti, Stefania Baronio, Luca Floreano, Luca Schio, Erik Vesselli, Alberto Verdini

**Affiliations:** ^1^ Department of Physics and Geology University of Perugia Via A. Pascoli Perugia 06123 Italy; ^2^ Department of Physics University of Trieste Via Valerio 2 Trieste 34127 Italy; ^3^ CNR ‐ Instituto Officina dei Materiali Area Science Park Trieste 34149 Italy; ^4^ Centre for Energy Environment and Transport Giacomo Ciamician University of Trieste Via Valerio 6/1 Trieste 34127 Italy; ^5^ CNR‐IOM, Perugia Unit, c/o Department of Physics and Geology University of Perugia Via A. Pascoli Perugia 06123 Italy

**Keywords:** metalation, photoelectron spectroscopy, phthalocyanines, surface chemistry, x‐ray absorption spectroscopy

## Abstract

Phthalocyanines (Pcs) are essential organic molecules with wide‐ranging applications in fields such as catalysis and optoelectronics, owing to their stable aromatic structure and exceptional thermal stability. Equally important is their versatility, which can be achieved by modifying the central metal atom. In this context, metalation—the process by which a central metal atom is incorporated into the macrocycle—plays a critical role in tailoring both the electronic and structural properties of Pcs. In this work, we investigate the thermally activated on‐surface synthesis of palladium phthalocyanine (PdPc) by depositing metal‐free phthalocyanine ($H_{2}\mathrm{Pc}$) onto a Pd(001) surface in ultra‐high vacuum (UHV) conditions. Using temperature‐dependent X‐ray photoemission spectroscopy (XPS), we analyze the evolution of the N 1s core level signal, confirming the successful formation of PdPc. The appearance of a secondary peak in the Pd 3d core level, attributed to Pd atoms coordinated within the phthalocyanine molecule after metalation, provides further evidence for the PdPc synthesis. Additionally, near‐edge X‐ray absorption fine structure (NEXAFS) reveals that no desorption or chemical degradation occurs during the process. We believe this study represents a significant step forward in the scalable synthesis of PdPc, which, like other Pd‐containing organic molecules, holds great potential for applications in catalysis and cancer therapy.

## Graphical abstract

Surfaces enable scalable reactions. Here, Pd(001) drives the self‐metalation of $H_{2}\mathrm{Pc}$, where the molecule captures a substrate atom to form PdPc. Spectroscopy reveals the reaction pathway, marking the first demonstration of phthalocyanine self‐metalation on Pd.

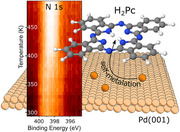

## 1 Introduction

Phthalocyanines (Pcs) are organic compounds widely exploited in technologically relevant materials for numerous applications, including dye‐sensitized solar cells^[^ ^1^ ^]^ and oxidation catalysis.^[^ ^2^ ^]^ Additionally, they serve as key components in industrial pigment production.^[^ ^3^ ^]^ These applications benefit from the excellent thermal stability of Pcs and their band gap in the visible range, which make them highly suitable for advancing various technologies.^[^ ^4^ ^]^

Furthermore, their planar geometry, characterized by a π‐conjugated electronic structure similar to that of porphyrin compounds, enables self‐assembly via in‐plane interactions through external functional groups, facilitating structural order and coordination.^[^ ^3^ ^]^ Beyond this, the planar structure keeps the center of the molecule relatively close to the supporting surface, allowing the surface to actively participate in key processes, such as metalation or charge transfer mechanisms, which underlie the surface trans‐effect.^[^ ^5^, ^6^, ^7^ ^]^

Metalation involves the coordination of a metal atom with the Pc molecule through the pyrrolic nitrogen atoms in its central ring.^[^ ^8^, ^9^, ^10^ ^]^ On‐surface metalation (by pre‐ or post deposition of metals), self‐metalation (when the metal atoms originate from the substrate), and trans‐metalation (when the macrocycle exchanges its metal center with another metal atom) have been mostly studied for the case of porphyrins.^[^ ^11^, ^12^, ^13^ ^]^ Notably, the presence of oxygen can facilitate the process, as demonstrated for metal‐free porphyrins on Pd(100), where self‐metalation is promoted under near‐ambient conditions.^[^ ^14^ ^]^ All of them may be regarded as a viable route to the synthesis of highly pure and oriented layers of tetrapyrroles coordinated to metals of almost free choice. The mechanism of nitrogen chelation and corresponding hydrogen release requires overcoming an energy barrier that depends on the specific substrate, metal atom, and molecular conformation. For example, in the case of $H_{2}\mathrm{Pc}$ on Ag(111), metalation with co‐adsorbed iron atoms is nearly complete at room temperature.^[^ ^15^ ^]^ In contrast, self‐metalation with Ag atoms from the substrate does not occur spontaneously and must be induced by external triggers, such as manipulation with a scanning tunneling microscope (STM) tip.^[^ ^16^ ^]^

Studies on substrates with different electronic properties, such as semi‐metal Sb(111) and Bi(111) surfaces, reveal that self‐metalation of empty Pc molecules completes at 470 and 670K, respectively.^[^ ^17^ ^]^ Similarly, a Cu(111) surface induces the conversion of $H_{2}\mathrm{Pc}$ into both deprotonated $H_{0}\mathrm{Pc}$ and metalated CuPc molecules at temperatures above room temperature.^[^ ^10^ ^]^ Thus, the process can be promoted or tuned in different ways, including also the chemical potential from the gas phase.^[^ ^14^ ^]^

Despite the formation of stable metal– organic complexes with various metals from the periodic table, the synthesis of such complexes via on‐surface metalation (or self‐metalation) remains underexplored for many systems and substrates.

In this work, we report the self‐metalation of metal‐free phthalocyanines by surface palladium atoms. Although significant challenges remain, this process holds potential for scalability and industrial applications, making this study an initial step in assessing its viability. Moreover, our motivation stems from the remarkable properties of metal–organic compounds such as phthalocyanines and tetrapyrroles. Notably, Pd‐based organic molecules exhibit exceptional catalytic activity, making them invaluable in industrial and green chemistry.^[^ ^18^, ^19^ ^]^ Their coordination chemistry also enables the development of highly sensitive sensors^[^ ^20^, ^21^ ^]^ and efficient photovoltaic materials.^[^ ^1^ ^]^ Furthermore, Pd‐based compounds have garnered significant interest in photodynamic cancer therapy, where their interaction with light enhances therapeutic efficacy.^[^ ^22^ ^]^

Using a spectroscopy‐based approach, we characterize the self‐metalation mechanism as a function of temperature for one monolayer of $H_{2}\mathrm{Pc}$ deposited on a Pd(001) substrate in ultra‐high vacuum (UHV) conditions. In particular, the system was studied by synchrotron‐based X‐ray photoemission spectroscopy (XPS), which allowed tracking the metalation process as a function of the annealing temperature, also furnishing an estimation for the activation energy. Near‐edge X‐ray absorption fine structure (NEXAFS) analysis confirms that the molecules neither desorb nor decompose upon annealing within the temperature range used in the experiment.

## 2 Results and Discussion

**Figure 1:**
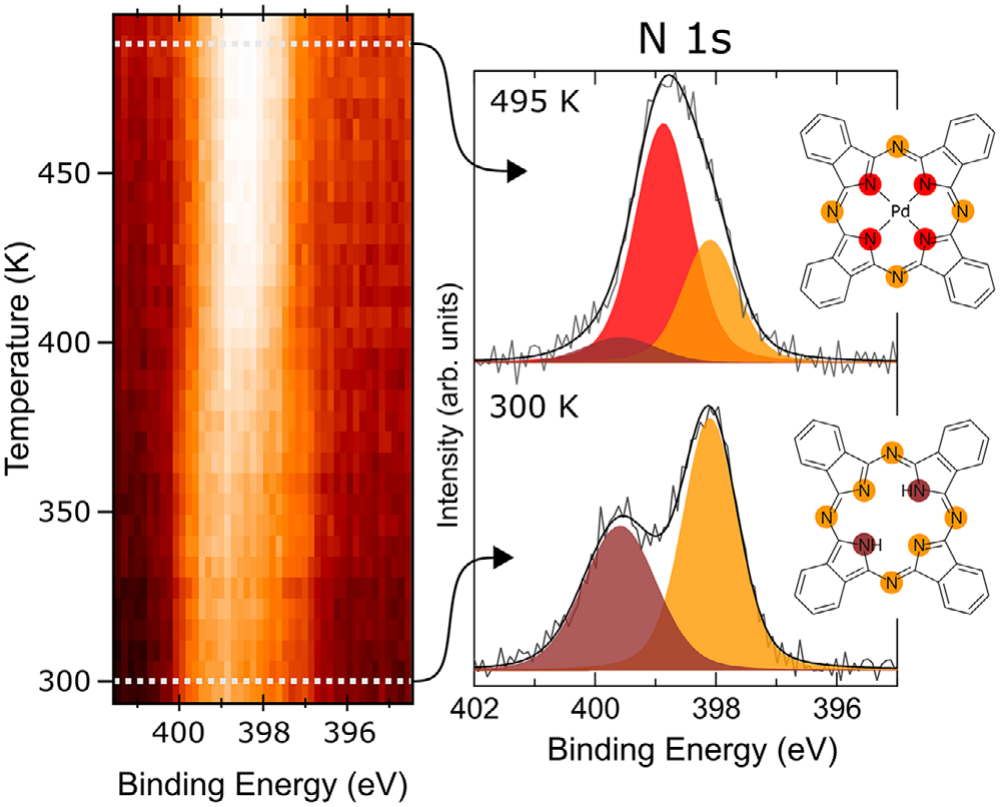
Temperature‐dependent N 1s XPS map. Bright areas correspond to higher signal intensity. The white dotted lines mark the temperatures at which the two high‐resolution spectra shown on the right were recorded, specifically at 300 and 495K. In these spectra, the black solid line represents the best‐fit envelope, and the filled curves correspond to the peak deconvolution. To the right of the spectra, sketches of the $H_{2}\mathrm{Pc}$ and PdPc molecules are displayed, with the N atoms highlighted in colors matching the corresponding components of the XPS spectra deconvolution.

The system under investigation consists of a monolayer of $H_{2}\mathrm{Pc}$ grown on the clean Pd(001) termination. In Figure 1, the plot on the left shows the temperature‐dependent evolution of the XPS signal in the N 1s binding energy region. The temperature of the sample was linearly increased at rates of 0.07 and $0.2\;Ks^{-1}$, in the temperature ranges from 300 to 335K and from 335K to 500K, respectively, while the XPS spectra were continuously recorded. From the plot, a temperature‐dependent evolution of the N 1s peak can be noticed. The figure also includes two highly resolved N 1s XPS spectra recorded at a low (300K, as deposited) and high (495K, after the annealing ramp) temperatures, visible on the right‐hand side of Figure 1. These high‐resolution spectra are presented along with their best‐fit envelopes and deconvolution profiles, aiming to resolve the initial and final states of the system.

It should be noted that the stoichiometric ratio of pyrrolic to aminic nitrogen deviates from the expected 1:3 value, which can be attributed to photoelectron diffraction effects altering their relative intensities by up to 50%.^[^ ^23^ ^]^ This interpretation is further supported by the NEXAFS spectra presented below, which confirm that the molecules remain intact and do not fragment into subunits.

At low temperature, the N 1s profile is well modeled by a convolution of two peaks, centered at 399.6 and 398.1eV, and commonly associated with the two pyrrolic (─NH─) and the six aminic (═N─) N atoms present in the $H_{2}\mathrm{Pc}$ molecules, respectively,^[^ ^15^, ^24^ ^]^ as sketched in the inset of Figure 1, proving that no metalation occurs at room temperature.

Considering the N 1s spectra obtained after the temperature ramp at 500K, a third spectral component emerges. Although the peaks associated with the pyrrolic and aminic N atoms remain at the same binding energies, albeit with lower intensity, an additional feature appears at 398.9eV. This new peak indicates the presence of the metalated phthalocyanine (PdPc).

Indeed, the metalated molecule features four inner N atoms coordinated to the central Pd atom (as illustrated in Figure 1), which contribute to the same XPS signal due to their chemical equivalence. Conversely, the four bridging aminic N atoms remain largely unaffected by the metalation process, and their contribution persists in the spectrum, although with reduced intensity. A similar evolution of the N 1s XPS core level has been reported in analogous systems and attributed to the metalation process.^[^ ^9^, ^17^ ^]^

It is important to note that the metalation is incomplete at 495K, with 84±12% of metalated molecules, as evidenced by a residual contribution from the pyrrolic N atoms, which are characteristic of the $H_{2}\mathrm{Pc}$ molecules. Incomplete metalation may stems from kinetic hindrance effects, associated with the diffusion of ad‐atoms toward the center of the molecules' islands, as observed, for example, in the case of the metalation of metal–organic coordination networks by metal ad‐atoms.^[^ ^25^ ^]^ However, using higher temperatures was avoided, as structural degradation of the organic framework and/or desorption from the surface may set in.

**Figure 2:**
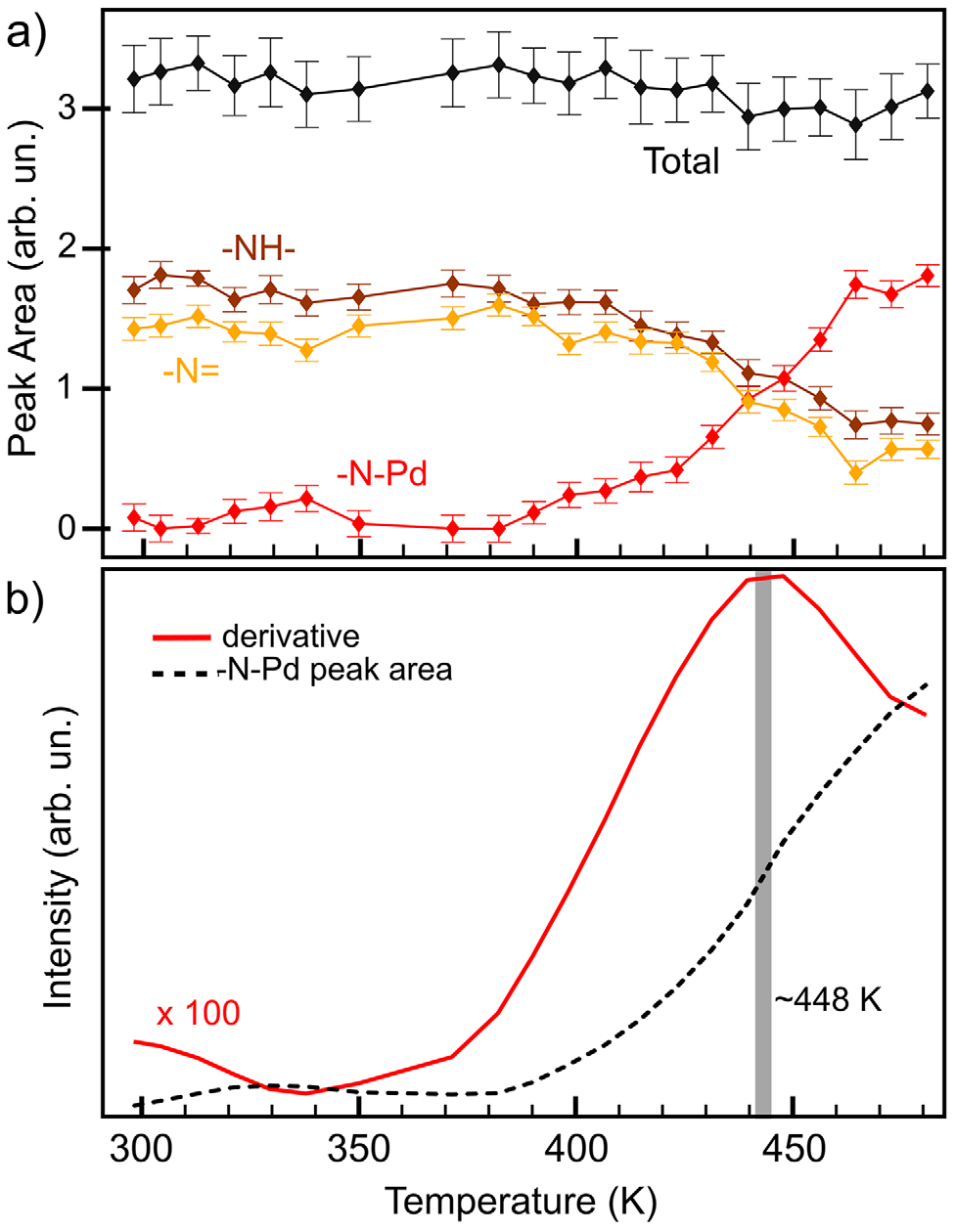
(a) Intensity of the N 1s peak components as a function of the temperature and their total sum. (b) The ─N─Pd intensity as a function of temperature, after the Gaussian smoothing process, in black, together with its first derivative, in red. The temperature for which the derivative is maximum is highlighted in gray.

Figure 2a shows the intensities of the N 1s core‐level components, extracted from fitting the spectra comprising the XPS map in Figure 1, as a function of the sample temperature, along with their total sum. Notably, the total sum remains constant within the uncertainty throughout the temperature ramp, indicating that no molecular desorption occurred during the annealing process. Conversely, the intensities of the aminic (─NH─) and pyrrolic (─NH─) peaks decrease, while the intensity of the metalated species (─N─Pd) increases, as expected.

Focusing on the ─N─Pd component, which is specific to the metalated phthalocyanine, its peak intensity can be used to estimate the activation energy of the metalation process. To this end, Figure 2b shows the ─N─Pd peak intensity as a function of temperature, after Gaussian smoothing, along with its first derivative calculated with respect to time (which is proportional to the temperature variation via the annealing rate). Using the Redhead model,^[^ ^26^ ^]^ the activation energy $E_{a}$ can be estimated with the following formula:

(1) $$E_{a}=\left[\ln\left(\nu \frac{T_{m}}{k}\right)-3.64\right]RT_{m}$$

where $\nu =10^{13}\;s^{-1}$ is the standard pre‐exponential factor,^[^ ^27^ ^]^ R is the gas constant, $k=0.2\;Ks^{-1}$ is the annealing rate, and $T_{m}$ is the temperature at which the derivative reaches its maximum, specifically 448K. Using this model, the activation energy is calculated as $E_{a}=1.3\pm 0.3\;eV$, which is comparable to activation energies reported for similar processes.^[^ ^8^, ^28^ ^]^ Please note that, in principle, the Redhead formula is not applicable to this complex multistep reaction, which involves the loss of two protons and the addition of a Pd atom. Here, it is used solely to provide an estimate of the energy barrier. Additionally, the assumed prefactor may significantly deviate from the correct value and, accordingly, the error bar associated with the estimated value is as wide as 20%–25%. Nevertheless, we believe that even a rough yet reasonable estimate of the barrier can serve as a useful reference.

**Figure 3:**
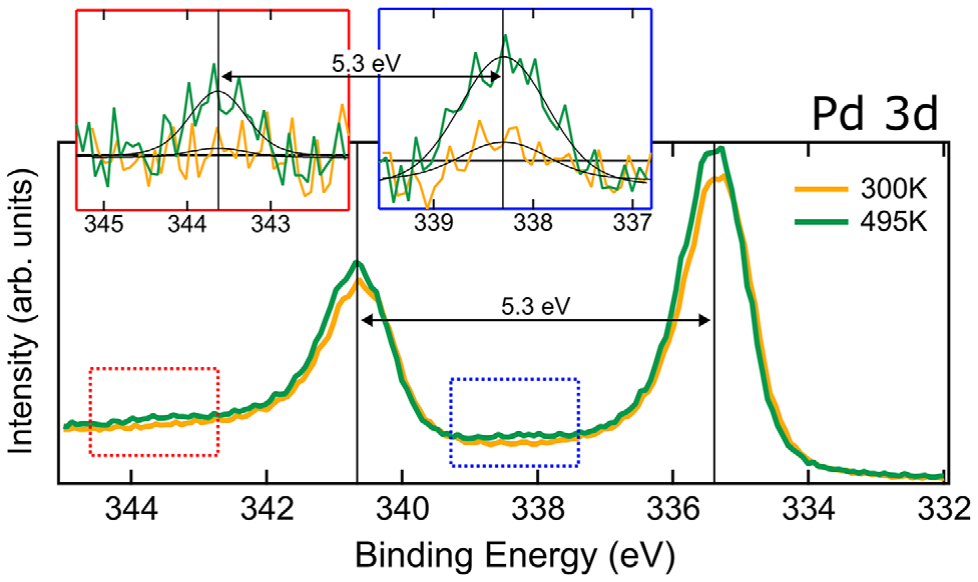
Pd 3d XPS spectra are shown for the as‐deposited sample (in yellow) and after the temperature ramp (in green). The insets provide a zoom of the areas indicated by the dotted rectangles in the main graph, highlighting the secondary features that emerge at 495K. In the insets, the black lines represent the Gaussian fit of the signal after background subtraction, which was performed using a polynomial fit of the 300K spectrum.

Further evidence confirming the occurrence of metalation is provided by the XPS Pd 3d spin‐orbit doublet, shown in Figure 3, measured before and after the temperature ramp. The main Pd 3d components, originating from the metal bulk and surface, remain unaffected by the sample heating.^[^ ^29^, ^30^ ^]^ However, a closer examination at higher binding energies reveals the emergence of a new doublet, shifted by approximately 2.5eV relative to the bulk peak at 335eV. To detect the small contribution of Pd incorporated into the Pc, we optimized the photon energy for the best compromise between photon flux and electron mean free path.

It has been demonstrated that the Doniach–Šunjić line shape is inadequate for modeling the Pd 3d core‐level profile,^[^ ^31^ ^]^ due to its unusually large asymmetry in the electronic structure.^[^ ^32^ ^]^ As a result, the level of accuracy required to completely remove the bulk and surface contributions from the spectra in Figure 3 cannot be achieved using such fitting functions. To better visualize the signal originating from the new components, the room‐temperature spectrum within the relevant energy range was modeled with a polynomial curve. This curve was then adapted to fit the 495K spectrum, allowing for the removal of the signal contributions from the substrate.

The insets of Figure 3 show the difference between the original spectra and the polynomial background. The new features appear at 338.3±0.1 and 343.6±0.1eV upon annealing at 495K, as revealed by a Gaussian fit of the spectra in the insets. Although the signal is relatively weak, the spin‐orbit separation is consistent with the Pd 3d doublet.^[^ ^29^ ^]^ We attribute these new components to Pd atoms embedded in the phthalocyanine macrocycle as it undergoes metalation. The binding energy of 338.3eV is in good agreement with the values reported for PdTPP^[^ ^33^ ^]^ and PdPc,^[^ ^34^ ^]^ confirming that mild annealing successfully metalates the $H_{2}\mathrm{Pc}$ monolayer. However, the number of Pd atoms in the molecules is very low compared to the surface and bulk Pd atoms (less than 1% of a monolayer), which explains the weakness of the signal.

Additional insights into the process can be obtained from the C 1s signal, plotted in Figure S1. The C 1s profile retains its shape and energy position during the annealing process, indicating that no significant damage occurred and, once again, no desorption took place.

**Figure 4:**
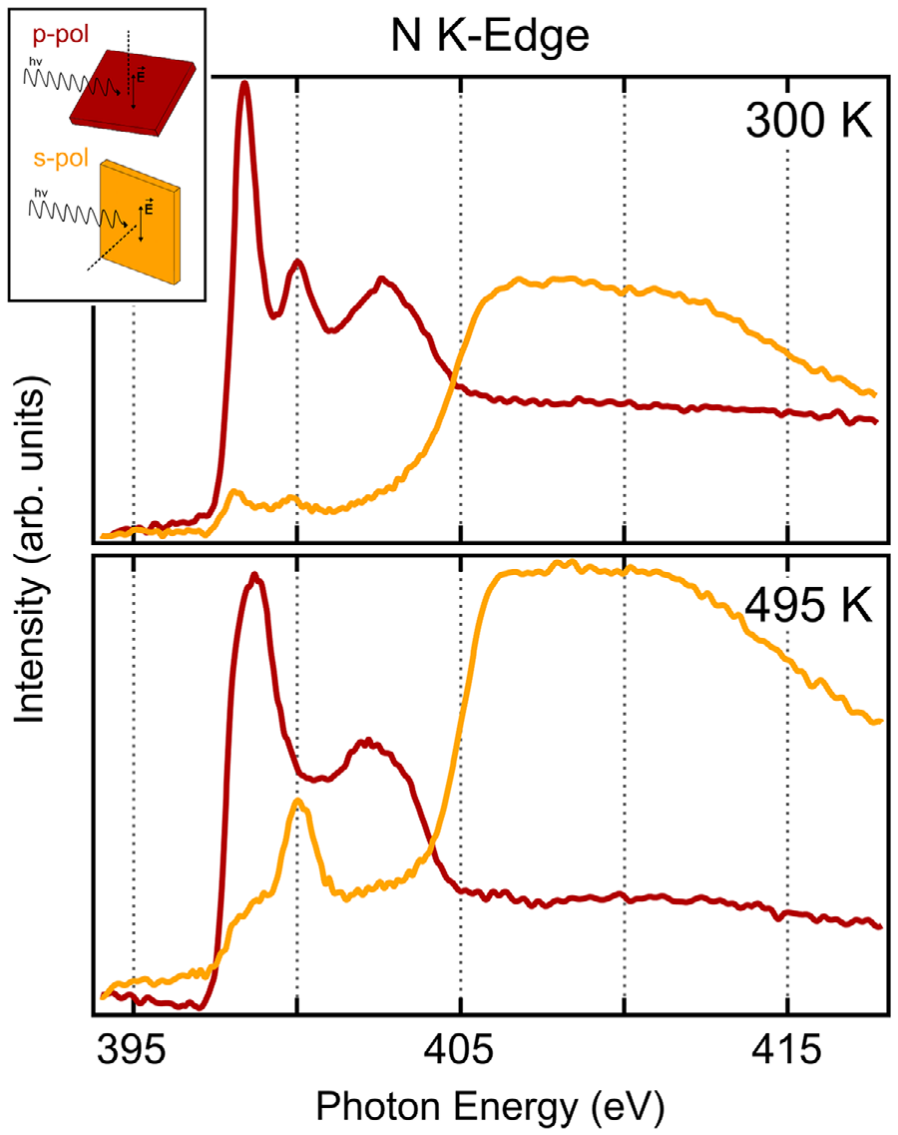
N K‐edge spectrum before (300K) and after (495K) the annealing ramp. The inset shows the p and s polarization geometries, corresponding to the red and yellow spectra, respectively.

**Figure 5:**
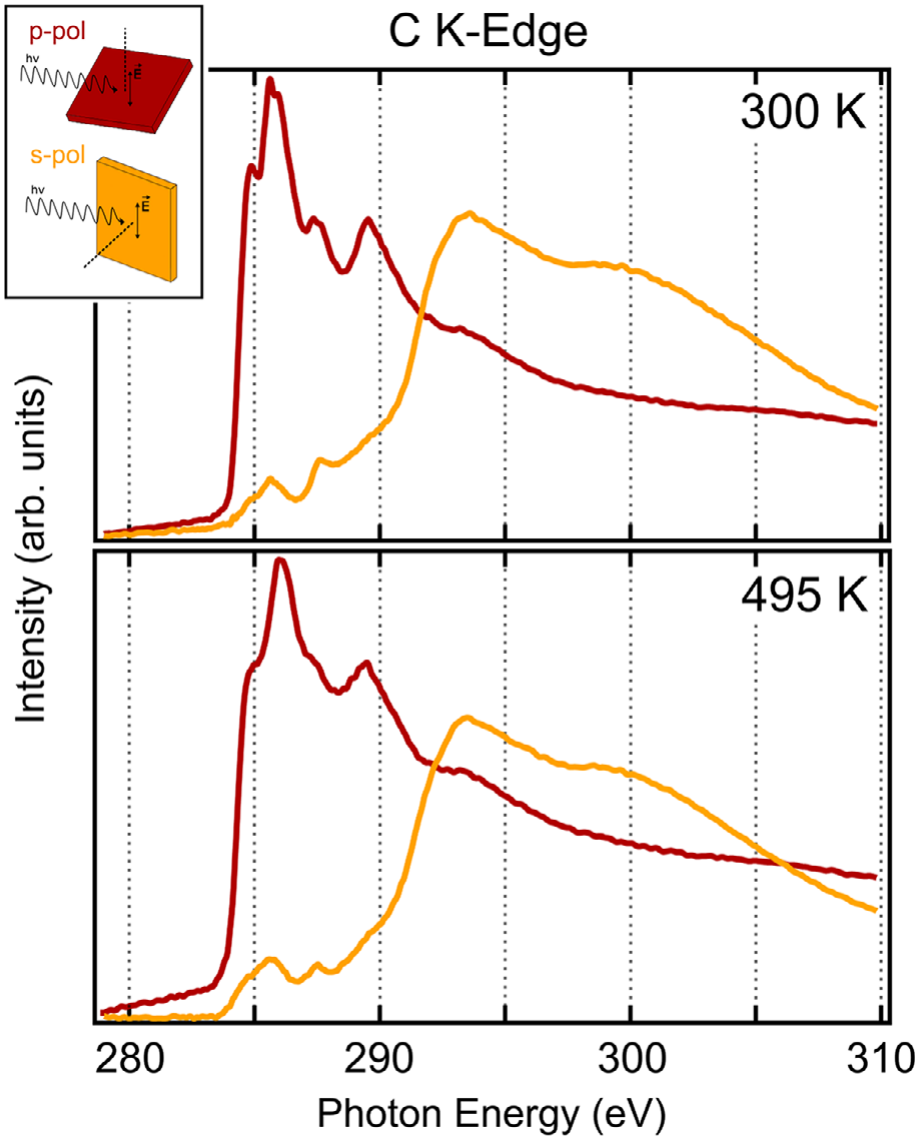
C K‐edge spectrum before (300K) and after (495K) the annealing ramp. The inset shows the p and s polarization geometries, corresponding to the red and yellow spectra, respectively.

N and C K‐edge NEXAFS spectra were collected before and after the temperature ramp to investigate the geometric alignment of the molecular moieties and the evolution of the bonding and anti‐bonding electronic states. The spectra are shown in Figures 4 and 5, where in each case, we report the partial absorption yield signal for two different geometries, i.e., with the light linear polarization either normal (s‐polarization) or parallel (p‐polarization) to the plane of incidence, as shown in the inset sketch.

This approach is based on the fact that light absorption depends on the orientation of the electric field with respect to the geometry of the electronic orbitals involved in the process (dipole approximation). Therefore, information about the molecular geometry can be inferred from the orientation of the molecular bonds. In the present system, this is particularly useful for detecting the orientation of the directional π‐conjugated electronic orbitals, allowing us to check the planarity of the molecule.

The N K‐edge spectra of as‐deposited phthalocyanine closely resemble those reported for $H_{2}\mathrm{Pc}$ multilayers grown on various substrates. In spectra measured with *p*‐polarization, we identify three main features corresponding to transitions into $\pi^{*}$‐symmetry unoccupied molecular orbitals.^[^ ^35^ ^]^ When comparing these spectra to those of highly oriented $H_{2}\mathrm{Pc}$ multilayers,^[^ ^36^ ^]^ we observe that the broad feature around 402–402.5eV arises from the merging of two distinct resonances in this energy range. This effect is attributed to a slight charge transfer from the substrate in the case of a single monolayer on Pd(100). Furthermore, these three resonances nearly disappear in s‐polarization spectra, indicating that $H_{2}\mathrm{Pc}$ molecules adopt a nearly flat orientation on the Pd(100) surface.

After annealing to 495K, the NEXAFS spectra undergo significant changes beyond the effects of metal incorporation, indicating substantial charge transfer from the substrate to the phthalocyanine. To better analyze the spectra in the self‐metalated phase, we compare them with NEXAFS measurements of a highly oriented $H_{2}\mathrm{Pc}$ monolayer on graphene, as recently reported.^[^ ^37^ ^]^ Indeed, Pd and Ni in their oxidation state (II) are isovalent and exhibit high stability in a tetraplanar square coordination with nitrogen.^[^ ^38^ ^]^

The primary spectral change upon Pd incorporation is the emergence of a sharp $\sigma^{*}$‐symmetry resonance at low energy, located very close to the lowest‐energy $\pi^{*}$‐symmetry resonance.^[^ ^37^ ^]^ This state is the only peak clearly observed in the s‐polarization NEXAFS spectrum and corresponds to transitions into the Pd $4d_{x^{2}-y^{2}}$ atomic orbital. In p‐polarization, Pd incorporation results in only two main detectable resonances, suggesting a strong charge transfer. This transfer leads to LUMO filling and a corresponding shift to lower energy of the higher $\pi^{*}$‐symmetry resonances.^[^ ^39^ ^]^

The residual intensity observed in s‐polarization below the ionization threshold, spanning the 397–404eV range, does not exhibit specific features. This intensity could result from hybridization with the substrate or from a Fermi step effect due to the molecule's close proximity to the surface.^[^ ^40^ ^]^ Despite these changes, the NEXAFS spectra retain strong linear dichroism after annealing to 495K, confirming that $H_{2}\mathrm{Pc}$ remains oriented parallel to the surface.

The C K‐edge NEXAFS spectra, both before and after annealing to 495K, exhibit behavior similar to that of the N K‐edge. The carbon spectra are characterized by an additional resonance at the lowest energy (LUMO), associated with transitions into the $\pi^{*}$‐symmetry state localized on the outer aromatic rings of the four isoindole groups.^[^ ^41^ ^]^ This LUMO appears as a distinct shoulder of the most intense resonance, LUMO+1, which is localized on the inner pyrrolic rings and corresponds to the leading resonance in the N K‐edge spectra. Compared to spectra measured for $H_{2}\mathrm{Pc}$ multilayers,^[^ ^35^ ^]^ the most significant difference is the quenching of the LUMO+1 resonance, attributed to charge transfer from the substrate. This observation is consistent with the previously discussed N NEXAFS spectra. The linear dichroism at the C K‐edge is also in agreement with that observed at the N K‐edge.

Upon annealing, the C spectra show a broadening of all $\pi^{*}$‐symmetry resonances, along with an increase in residual intensity below the ionization threshold, particularly in s‐polarization. This residual intensity appears as an unresolved constant background superimposed on the $\pi^{*}$‐symmetry resonances, similar to what is observed in the N K‐edge spectra.

## 3 Conclusion

We reported the self‐metalation of $H_{2}\mathrm{Pc}$ molecules deposited on a Pd(001) substrate, occurring within a temperature window between room temperature and 500K, with the highest conversion rate observed around 440K. At room temperature, no evidence of metalation is found, while at 500K, approximately 84% of the molecules are metalated. Notably, the metalation is further confirmed by the XPS Pd 3d core‐level signal, where a new component appears, corresponding to the palladium atoms embedded in the molecular macrocycle upon metalation, and also supported by NEXAFS. Throughout the metalation process, we observe no desorption up to 500K, nor any chemical decomposition of the peripheral moieties.

## 4 Experimental

The experiment took place at the ALOISA beamline of the Italian CNR at the Elettra synchrotron radiation facility in Trieste (Italy). Preparation and analysis of the samples were carried out in UHV conditions, with a base pressure better than $5\times 10^{-10}$ mbar. The Pd(001) single crystal (SPL – Surface Preparation Lab.) was prepared by repeated cycles of sputtering (3kV, 6mA, Ar^+^) and annealing (1000K). Reflection high‐energy electron diffraction (RHEED) was used to check the surface crystalline quality, while the absence of contaminants was proven by means of XPS (checking the C, N, and O 1s core levels). The $H_{2}\mathrm{Pc}$ molecules were sublimated from a resistive heated crucible at 610K on the sample kept at room temperature. The film thickness was estimated using a quartz microbalance through calibration and monitoring of the molecular deposition rate. NEXAFS spectra were taken in partial yield mode employing a linearly polarized (95%) photon beam. Keeping the photon impinging grazing angle fixed to $6^{\circ }$, the sample was rotated around the photon beam axis to change the scattering geometry from (close‐to) *s*‐polarization to *p*‐polarization, i.e., with the light polarization plane perpendicular or parallel to the plane of incidence, respectively. The absorption spectra were normalized to spectra measured on the clean substrate before molecular deposition. The drain current on the last refocusing Au‐coated mirror of the beamline was collected synchronously to the NEXAFS to take into account time‐ and energy‐dependent intensity fluctuations of the light beam. The same drain current signal was used for the absolute calibration of the C and N ionization thresholds, as those elements are naturally present in the optical elements of the beamline. More details about the NEXAFS setup can be found elsewhere.^[^ ^42^ ^]^ XPS spectra were taken using a photon energy of 525eV for the N and C 1s core levels, 430eV for the Pd 3d core level. A Voigt profiles were adopted for fitting the molecular N 1s core levels that are not affected by the non‐adiabatic asymmetric tail. For the XPS N 1s spectra, both from the temperature ramp and the high‐resolution measurements, a linear background was subtracted during fitting.

## Conflict of Interests

The authors declare no conflict of interest.

## Supporting information

Supporting Information

## Data Availability

Research data are not shared.
