# Supplementary material for: Thermally Activated on‐Surface Self‐Metalation of Pd‐Phthalocyanines
Source: Chemistry. 2025 Apr 18;31(28):e202500944. doi: 10.1002/chem.202500944 (PMC12089924; doi:10.1002/chem.202500944)
Supplement: Supplementary file 1 — Supporting Information [file CHEM-31-e202500944-s001.docx]

**Supplementary Information**

**“Thermally Activated On-Surface Self-Metalation of Pd-Phthalocyanines”**

Mattia Bassotti,^*[a]^ Stefania Baronio,^[b]^ Luca Floreano,^[c]^ Luca Schio,^[c]^ Erik Vesselli,^[b],[c],[d]^ and Alberto Verdini.^[e]^

[a] M. Bassotti*

Department of Physics and Geology, University of Perugia, Via Alessandro Pascoli snc, 06123 Perugia (Italy)

E-mail: bassottimattia@gmail.com

[b] Dr. S. Baronio, Prof E. Vesselli

Department of Physics, University of Trieste, Via Valerio 2, 34127 Trieste (Italy)

[c] Dr. L. Floreano, Dr. L. Schio, Prof E. Vesselli

CNR - Instituto Officina dei Materiali, Area Science Park, 34149 Trieste (Italy)

[d] Prof. E. Vesselli

Centre for Energy, Environment and Transport Giacomo Ciamician, University of Trieste, Via Valerio 6/1, 34127 Trieste (Italy)

[e] Dr. A. Verdini

CNR-IOM, Perugia Unit, c/o Department of Physics and Geology, University of Perugia, Via A. Pascoli, 06123 Perugia (Italy)


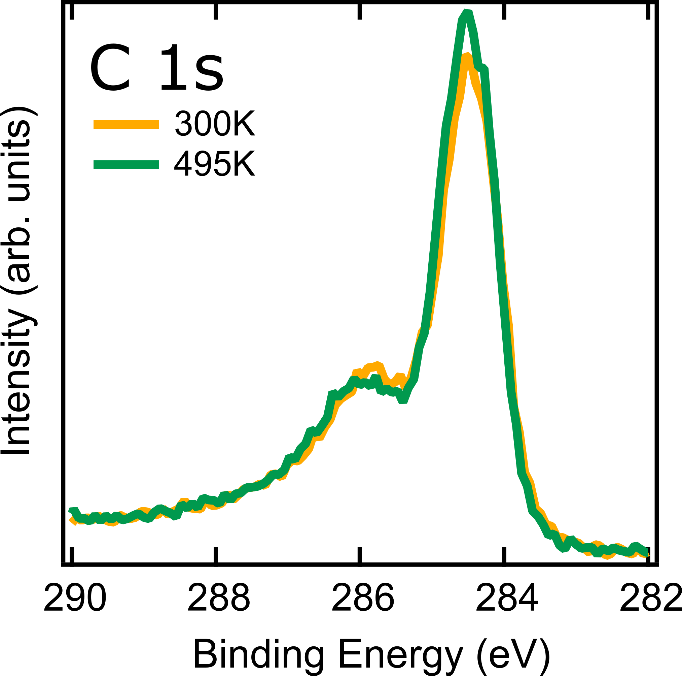


**Figure S1.** C *1s* XPS spectra for the pristine (300 K) and post annealing (495 K) H_2_Pc deposited on Pd(001). The comparison between the spectra witnesses that no desorption occurred during the temperature ramp.
